# Immune microenvironment dynamics in pregnant patients with concomitant autoimmune diseases: mechanisms, challenges, and clinical significance

**DOI:** 10.1016/j.mmr.2026.100015

**Published:** 2026-04-04

**Authors:** Zi-Jun Ma, Jun Chen, Chen-Xi Yang, Shun-Ping Lin, Quan Gong, Zachary Braunstein, Ying-Ying Wei, Xiao-Quan Rao, Ji-Xin Zhong

**Affiliations:** a Department of Rheumatology, Fujian Medical University Union Hospital, Fuzhou 350001, China; b Division of Rheumatology and Immunology, Department of Internal Medicine, Tongji Hospital, Tongji Medical College, Huazhong University of Science and Technology, Wuhan 430030, China; c Sinopharm Dongfeng General Hospital (Hubei Clinical Research Center of Hypertension), Hubei Key Laboratory of Wudang Local Chinese Medicine Research, Hubei University of Medicine, Shiyan 442000, Hubei, China; d Fujian Institute of Clinical Immunology, Fuzhou 350001, China; e Department of Immunology, School of Medicine, Yangtze University, Jingzhou 434023, Hubei, China; f Division of Hematology, Department of Internal Medicine, James Comprehensive Cancer Center, the Ohio State University Wexner Medical Center, Columbus, OH 43210, USA; g Division of Cardiology, Department of Internal Medicine, Tongji Hospital, Tongji Medical College, Huazhong University of Science and Technology, Wuhan 430030, China; h Key Laboratory of Vascular Aging (HUST), Ministry of Education, Wuhan 430030, China

**Keywords:** Autoimmune diseases (AIDs), Pregnancy, Immune microenvironment, Maternal-fetal immune adaptation, Clinical management

## Abstract

Throughout pregnancy, the immune microenvironment undergoes dynamic changes in patients with concomitant autoimmune diseases (AIDs). These alterations not only affect disease activity and clinical manifestations, but also play a pivotal role in sustaining maternal-fetal immune tolerance and pregnancy outcomes. Extensive preclinical studies have elucidated the mechanisms of immune regulation in normal pregnancy, whereas the dynamic immune changes in pregnancies complicated by AIDs remain poorly understood. Recent studies have revealed significant variations in immune responses to pregnancy among individuals with AIDs, which may contribute to distinct patterns of AIDs flares during this period. Despite substantial progress in immunology and reproductive medicine, comprehensive reviews addressing the dynamic changes in the immune microenvironment during pregnancy in the context of AIDs are lacking. In this review, we summarize existing knowledge and incorporate recent multidisciplinary findings, focusing on the dynamic changes in systemic immune adaptation and maternal-fetal immune interactions in the context of AIDs during pregnancy. We emphasize the clinical significance of these immune dynamics for optimizing management and therapeutic strategies. Additionally, we propose new perspectives and provide recommendations to guide future research and the development of personalized treatment approaches.

## 1 Background

Autoimmune diseases (AIDs) are characterized by disrupted immune homeostasis, a disorder that is particularly evident in women of reproductive age [1]. Accumulating evidence indicates that pregnant women with AIDs face a significantly increased risk of adverse pregnancy outcomes (APOs) [2]. During pregnancy, both systemic immune changes and localized immune adaptations at the maternal-fetal interface, especially those regulating the placental microenvironment, are essential for sustaining maternal-fetal immune tolerance and promoting favorable pregnancy outcomes [3]. In patients with AIDs, particularly autoimmune rheumatic diseases, gestation is associated with dynamic shifts in disease activity. Importantly, gestational immune adaptation is heterogeneous across different autoimmune disorders, which may underlie distinct, disease-specific patterns of flares and remission [4], [5]. While significant advances have been achieved in elucidating normal pregnancy immunoregulatory processes [6], [7], essential scientific issues remain, especially concerning the intrinsic differences in gestational immune modulation between AID-affected pregnancies and normal pregnancies, as well as disease-specific immune dynamics across various autoimmune disorders. Within this context, we systematically integrate recent multidisciplinary evidence to delineate the sequential and dynamic alterations of maternal-fetal immune regulation across pregnancy stages, clarify the interplay between disease-specific immune profiles and pregnancy immune adaptation, and underscore the significance of clinical management and treatment strategies in AIDs during pregnancy.

## 2 Changes in the immune microenvironment during pregnancy

### 2.1 Systemic immune microenvironment

During normal pregnancy, the maternal immune system undergoes finely tuned adaptive changes aimed at maintaining fetal tolerance. The complement system exhibits a dynamic balance, with increased activation counterbalanced by elevated inhibitory factors, providing anti-infective protection while avoiding fetal injury [8]. Concomitantly, granulocyte dynamics shift markedly, with neutrophil counts progressively increasing throughout pregnancy, accompanied by elevated levels of hematopoietic factors such as granulocyte colony-stimulating factor (G-CSF) and granulocyte-macrophage colony-stimulating factor (GM-CSF) [9], [10], yet their function can be altered. Although resting neutrophils show increased activity, upon activation, their oxidative burst and reactive oxygen species production capacity decrease [11]. These adaptations are likely crucial for sustaining immune tolerance and preventing harmful immune reactions directed against the fetus.

In parallel, monocytes exhibit dynamic changes throughout pregnancy. From early pregnancy, circulating monocyte counts progressively increase, marked by an increase in intermediate monocytes and a decrease in classical subsets [12], [13]. Concurrently, maternal monocytes show a shift toward proinflammatory subsets (e.g., increased IL-6^+^CD14^+^ or MIP-1α^+^CD14^+^ cells) together with increased reactive oxygen species production, indicating enhanced proinflammatory potential [14]. Conversely, in late pregnancy, monocytes adopt an anti-inflammatory phenotype, characterized by reduced cytokine production upon lipopolysaccharide stimulation compared with that in nonpregnant women, indicating a state of immune tolerance [15], [16]. This late-gestational monocyte hyporesponsiveness is conceptually reminiscent of sepsis-associated immunoparalysis, in which monocytes suppress proinflammatory responses to limit tissue damage [17], [18], [19]. This monocyte tolerance in late pregnancy likely serves as a critical immunoregulatory mechanism to preserve maternal immune homeostasis and prevent excessive immune activation [15]. In general, monocytes during pregnancy display characteristics of chronic low-grade inflammation and retain the capacity to restore immune function when appropriately stimulated, thus supporting immune balance between the mother and fetus.

Natural killer (NK) cells, which are vital components of the innate immune system, exhibit remarkable plasticity in their activity during pregnancy, enabling them to adapt to distinct local and systemic environments [20], [21]. In the peripheral blood, the numbers and activity of NK cells are generally reduced, which helps prevent fetal rejection [22]. In contrast, a notable increase occurs in both the quantity and activity of NK cells within the placental microenvironment [23]. This functional divergence between placental and circulating NK cells highlights the specificity and complexity of placental immune regulation.

T cells undergo key systemic adaptations during pregnancy to maintain maternal immune homeostasis. While total lymphocyte and T cell percentages remain stable, overall T cell numbers decline compared with those in the pre-pregnancy state [24]. Regarding subset distribution, CD4^+^ and CD8^+^ T cells within the αβ T cell population tend to decrease in early and late pregnancy, likely due to the markedly increased estrogen and progesterone levels characteristic of pregnancy [25]. Sex hormones modulate thymic function, thereby shaping T cell development and regulating peripheral T cell counts, which is essential for sustaining maternal immune balance [26]. Functionally, a dynamic shift occurs, a T helper (Th)1-dominant profile supports implantation in early stages, followed by Th2-mediated immune tolerance during mid-to-late pregnancy, with a re-establishment of Th1 predominance towards term to initiate labor [27], [28]. In addition, regulatory T cells (Tregs) play a pivotal role in maternal-fetal immune tolerance. During early pregnancy, peripheral Treg proportions markedly rise, enabling the suppression of autologous T cell proliferation and facilitating immune tolerance [29]. However, reports on Treg dynamics vary, likely due to inconsistent definitions, as both the CD25 and forkhead box P3 (FOXP3) markers are shared by activated T cells [30]. Accurate distinction is essential for assessing Treg fluctuations during pregnancy. Additionally, γδ T cells exhibit unique spatiotemporal distribution patterns. During the early phase of a normal pregnancy, γδ T cells increase in both the peripheral blood and decidua, predominantly comprising the Vδ1 T subset, which contributes to the establishment and maintenance of early pregnancy immune tolerance through secretion of anti-inflammatory cytokines and modulation of negative signaling pathways [31]. The Vδ2 T subset becomes predominant in the decidua during mid-pregnancy, whereas Vδ1 dominance returns at term [32]. In pathological pregnancies, γδ T cells may acquire proinflammatory and cytotoxic functions, warranting further investigation [33].

**Fig. 1:**
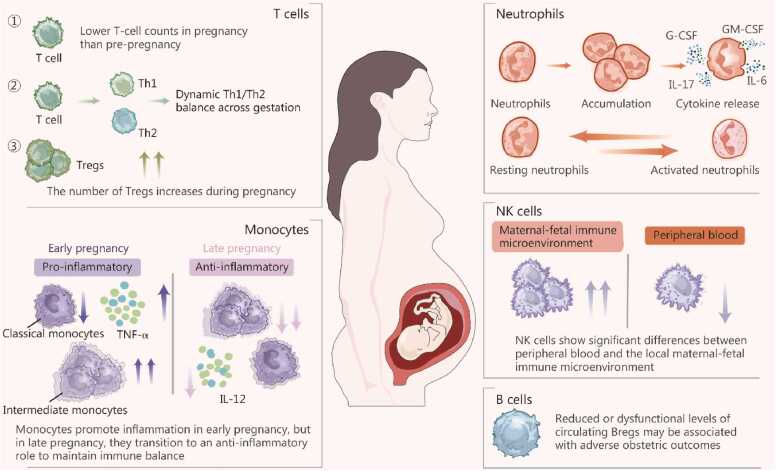
Alterations in the maternal systemic immune system in pregnancy. This figure illustrates systemic immune adaptations during normal pregnancy. Peripheral T cell counts decrease compared with the pre-pregnancy state, accompanied by a dynamic Th1/Th2 shift and an increase in Tregs. Neutrophils progressively accumulate and exhibit cytokine release in association with hematopoietic factors such as G-CSF and GM-CSF, with changes between resting and activated states. Monocytes show stage-dependent functional polarization, shifting from a proinflammatory profile toward an anti-inflammatory/tolerant profile in late pregnancy. NK cells display differences between peripheral blood and the local maternal-fetal immune microenvironment. In addition, reduced or dysfunctional circulating Bregs may be associated with adverse obstetric outcomes. Th1. T helper 1; Th2. T helper 2; Tregs. Regulatory T cells; IL-17. interleukin-17; IL-6. Interleukin-6; G-CSF. Granulocyte colony-stimulating factor; GM-CSF. Granulocyte-macrophage colony-stimulating factor; TNF. Tumor necrosis factor; IL-12. Interleukin-12; NK. Natural killer; Bregs. Regulatory B cells.

Although the significance of B lymphocytes in pregnancy has not been fully clarified, existing studies suggest that they play an active role during pregnancy [34], [35]. In recent years, regulatory B cells have been proposed as key participants in immune tolerance. Studies have shown that a reduction or dysfunction of circulating regulatory B cells may be associated with adverse obstetric outcomes [36], [37], as these cells are crucial in fostering the immunological milieu required for implantation and may also help re-establish fetal immune tolerance in immune-mediated pregnancy complications. Fig. 1 illustrates the dynamic changes in the maternal systemic immune microenvironment during normal pregnancy, including alterations in the quantity and function of key immune components such as neutrophils, monocytes, NK cells, T cells, and B cells.

### 2.2 Maternal-fetal immune adaptation

The endometrium (decidua) plays a key role in embryo implantation and pregnancy maintenance, with decidual immune cells being key regulators of these processes [38]. Decidual NK cells (dNK) constitute the predominant leukocyte population (50%—70%) in the decidua. Unlike peripheral blood NK cells, dNK cells exhibit minimal cytotoxicity and a functional bias toward immune regulation and angiogenesis, playing a crucial role in placental formation and fetal development [39]. During early pregnancy (8—10 weeks), dNK cells secrete angiogenic factors that promote spiral artery remodeling and optimize placental perfusion. With the progression of pregnancy, after 12—14 weeks of gestation, dNK cells mainly secrete cytokines [e.g., interleukin (IL)-8 and IL-10], which promote extravillous trophoblast (EVT) invasion and enhance placental function by modulating matrix metalloproteinase (MMP)-9 and inhibiting EVT apoptosis [40]. Additionally, dNK cells limit excessive trophoblast invasion and protect maternal tissues by transforming growth factor (TGF)-β and interferon (IFN)-γ secretion, maintaining immune tolerance and placental homeostasis [41].

Macrophages also contribute significantly to the decidual immune microenvironment. While scarce in the nonpregnant endometrium, macrophage numbers rise significantly in the decidua during pregnancy, driven by chemokine-mediated recruitment. Most exhibit an alternatively activated macrophage (M2) phenotype, expressing CD163, CD206, IL-10, IL-6, tumor necrosis factor (TNF), and chemokine C-C motif ligand (CCL)-4, a profile resembling that of macrophages stimulated by granulocyte-macrophage colony-stimulating factor and IL-10 [6]. During placentation, macrophages localize near invading trophoblasts and uterine spiral arteries and support trophoblast invasion and spiral artery remodeling by secreting and regulating MMPs, which promote extracellular matrix degradation and tissue remodeling [42]. Furthermore, decidual macrophages are crucial for maintaining immune homeostasis at the maternal-fetal interface. They not only mediate tissue clearance by phagocytosing apoptotic cells and debris but also help constrain excessive maternal immune responses through the secretion of anti-inflammatory cytokines such as IL-10 and TGF-β. Notably, macrophages within the placental bed express inhibitory receptors that interact with HLA-G expressed on extravillous trophoblasts. The interaction between HLA-G and these macrophages reprograms their cytokine profile, fine-tuning their function to promote immune tolerance [43].

**Fig. 2:**
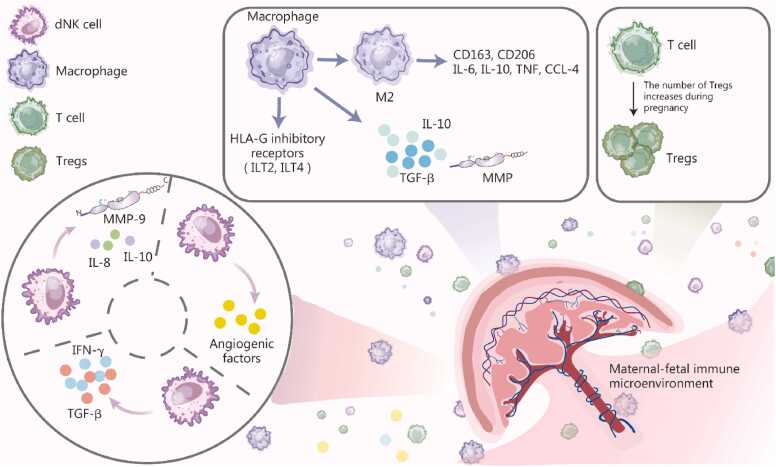
Maternal-fetal immune adjustment during normal pregnancy. This figure illustrates the dynamic immune microenvironment at the maternal-fetal interface during normal pregnancy. dNK cells, macrophages, and T cells constitute the major immune cell populations in the decidua. dNK cells secrete angiogenic factors, cytokines, and matrix metalloproteinases to support placental vascular remodeling. Decidual macrophages preferentially polarize toward an M2-like phenotype, expressing CD163 and CD206 and producing immunoregulatory mediators, thereby contributing to immune tolerance and tissue remodeling. Meanwhile, the proportion of Tregs increases during pregnancy, playing a central role in maintaining immune tolerance at the maternal-fetal interface. Together, these coordinated immune adaptations ensure successful implantation, placental development, and the maintenance of pregnancy. TNF. tumor necrosis factor; TGF. transforming growth factor; MMP. Matrix metalloproteinase; HLA. Human leukocyte antigen; CCL. Chemokine C-C motif ligand; dNK. Decidual natural killer; Tregs. Regulatory T cells; Th1. T helper 1; Th2. T helper 2; Th17. T helper 17; IL. Interleukin; ILT. Immunoglobulin-like transcript.

Decidual T cells, comprising 5%—20% of decidual lymphocytes in early pregnancy and increasing to 40%—80% at term, differ notably from peripheral blood T cells [44], [45]. Among CD4^+^ T cells, 10%—30% express FOXP3, indicating a significant enrichment of Tregs relative to the circulation [46]. The frequency of Th1 cells in the decidua moderately increases, whereas Th17 and Th2 cells are typically not enriched, indicating the presence of a mild inflammatory environment under the control of Tregs [47]. Decidual Tregs increase in early pregnancy, remain elevated during mid-pregnancy, and then decrease before delivery. Animal studies have demonstrated that T cells can interact with uterine NK cells, affecting the maternal hemodynamic response to pregnancy [48], [49]. The negative impact of uterine NK cell deficiency on decidual vascular remodeling is exacerbated by concurrent T cell deficiency. Insufficient or dysfunctional Tregs are linked to infertility, miscarriage, preeclampsia (PE), and fetal growth restriction [50]. Notably, unlike peripheral blood where CD4^+^ T cells predominate, at term pregnancy, CD8^+^ T cells constitute the major T cell subset in the decidua, with most being activated effector memory T cells; their low basal secretion of perforin and granzyme B results in reduced cytolytic activity [51]. Furthermore, the robust secretion of IFN-γ and other factors by decidual CD8^+^ T cells suggests a positive role in pregnancy, potentially contributing to vascular processes within the decidua [52]. Maintaining a proper equilibrium between immunotolerance and antiviral responses *via* decidual CD8^+^ cytotoxic T cells is essential for the normal course of pregnancy. Fig. 2 depicts the dynamic changes in the immune microenvironment at the maternal-fetal interface during normal pregnancy, highlighting dNK cells, macrophages, and T cells.

## 3 Autoimmune diseases during pregnancy

**Fig. 3:**
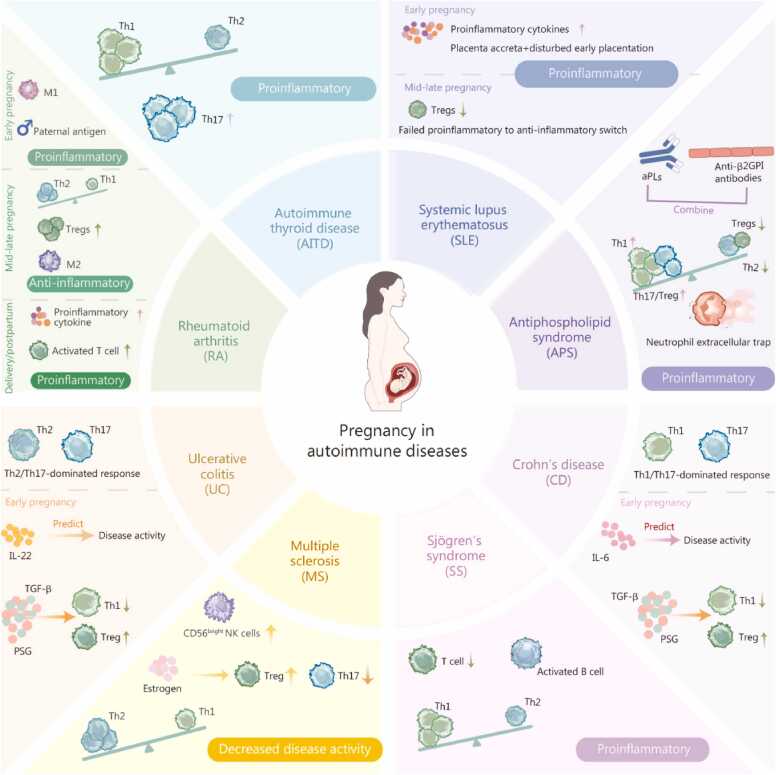
Immunological adaptations in autoimmune diseases during pregnancy. This figure summarizes disease-specific immunological adaptations across different autoimmune diseases during pregnancy. Pregnancy is associated with dynamic shifts in T cell-mediated immunity, including changes in Th1/Th2/Th17 balance and Treg abundance, which differentially influence disease activity. In conditions such as rheumatoid arthritis and multiple sclerosis, a shift toward anti-inflammatory immune profiles is commonly associated with disease improvement. In contrast, proinflammatory responses, enhanced Th17 activity, autoantibody production, or neutrophil extracellular trap formation may contribute to disease exacerbation in disorders such as systemic lupus erythematosus and antiphospholipid syndrome. TGF. Transforming growth factor; PSG. Pregnancy-specific glycoprotein; aPL. Antiphospholipid antibodies; Anti-β2GPI. Anti-β2-glycoprotein I antibodies; Tregs. Regulatory T cells; Th1. T helper 1; Th2. T helper 2; Th17. T helper 17.

The risk of APOs is markedly elevated in pregnant women with AIDs, driven not only by disease-specific immune dysregulation [e.g., anti-double-stranded DNA (dsDNA) antibodies in systemic lupus erythematosus (SLE) and inflammatory cytokines in rheumatoid arthritis (RA)], but also by environmental factors such as hormonal fluctuations and chronic systemic inflammation [53], [54], [55]. AIDs exhibit distinct patterns of immune adaptation at both the systemic and local levels, which highlights the clinical relevance of elucidating pregnancy-related immune microenvironmental shifts in these conditions to improve patient management. Fig. 3 illustrates a simplified diagram of immune alterations across common AIDs during pregnancy.

### 3.1 Systemic lupus erythematosus

SLE is a complex, chronic autoimmune disorder characterized by widespread immune-mediated tissue injury across multiple organ systems. Clinically, it presents with manifestations such as malar rash and positive antinuclear antibody [56]. The dysregulation of both innate and adaptive immune systems constitutes a significant driving force, with aberrant B cell activation and the resulting elevated autoantibodies [including anti-dsDNA, anti-smith, and antiphospholipid (aPL) antibodies] and immune complex deposition, leading to complement activation and tissue injury [57]. In addition, T cell imbalance, which is characterized by excessive Th17 activation and impaired Treg function, further disrupts immune regulation [58].

Women with SLE have a pregnancy rate approximately 30% lower than that of the general population, and face greater maternal risks (lupus flare, PE, and thrombosis) along with fetal and neonatal morbidity [59], [60], [61]. In SLE, higher disease activity during pregnancy is a key predictor of adverse outcomes [62]. Additionally, many immunosuppressive therapies pose risks [63]. Accordingly, clinical guidelines recommend conception only after achieving stable low disease activity under treatment with low-dose steroids and/or hydroxychloroquine (HCQ) [64].

Pregnancy involves complex immunoregulatory processes, posing significant challenges for patients with SLE. In healthy pregnancies, a shift from proinflammatory Th1/Th17 dominance to anti-inflammatory Th2/Treg dominance promotes maternal-fetal tolerance. In SLE patients, this regulatory shift is disrupted due to baseline immune dysregulation, increasing pregnancy-related risks. Early pregnancy in those with SLE is characterized by elevated levels of proinflammatory cytokines that impair embryo implantation and early placental development [65]. In the middle to late stages of pregnancy, SLE patients fail to transition from a proinflammatory to an anti-inflammatory state, resulting in insufficient anti-inflammatory responses. This may lead to a sustained proinflammatory milieu within the maternal immune system, ultimately increasing the probability of complications [66]. In healthy pregnancies, mid-to-late gestation is typically features by increased anti-inflammatory cytokines and expansion of Tregs to maintain maternal-fetal immune balance, but SLE patients exhibit impaired regulation, hindering this transition [65], [67]. Moreover, B cells in pregnant women with SLE show marked gene expression changes, including major histocompatibility complex pathway activation and inflammatory factor dysregulation, which, in conjunction with aberrant interactions with T cells and monocytes, synergistically promote disease progression [65], [67].

As mentioned above, the maternal immune system undergoes a transition from type 1 immunity to type 2 immunity during a healthy pregnancy, usually manifesting as an increased Th2-mediated immune response and a reduced Th1 response, thereby facilitating the sustenance of pregnancy. This immune shift may reduce disease activity in some SLE patients, resulting in “immunosuppression” rather than the typical “immune hyperactivation” phenotype. Mechanistically, a surge in estrogen facilitates Th2-mediated immune responses and inhibits Th1 responses, whereas progesterone further bolsters maternal-fetal immune tolerance by suppressing Th17 cell activity and increasing the ratio of Tregs [68]. Moreover, placental anti-inflammatory cytokines are crucial in maintaining local immune equilibrium by suppressing proinflammatory Th17 responses and enhancing the functionality of Tregs [69]. Furthermore, changes in galactosylation may play a role in the development of SLE. Research indicates that galactosylation-related transcripts are increasingly expressed in pregnant SLE patients, with their overall expression exceeding that of healthy individuals, implying that enhanced galactosylation-related immune characteristics may be present in SLE patients either at baseline or during gestation [70], [71]. Although the molecular mechanisms underlying immune modulation during normal pregnancy have been extensively documented, studies focusing on dynamic immune regulation in pregnant SLE patients are still exceedingly scarce, and further investigations are required to delineate the dynamic alterations in the immune microenvironment and to understand their direct effects on pregnancy outcomes.

In addition to the maternal systemic immune system, immune homeostasis within the maternal-fetal milieu is profoundly disrupted in SLE patients. Elevated aPL levels can lead to placental microangiitis and thrombosis, thereby increasing the risk of APOs [72]. Within the gestational uterus, dNK cells account for 70% of the maternal immune cell population, significantly contributing to placental development and immune equilibrium in the maternal-fetal milieu. Nevertheless, in pregnant individuals with SLE, dNK cells may exhibit functional abnormalities stemming from immune imbalance, resulting in unfavorable pregnancy outcomes [73]. However, current evidence suggests that a reduction in dNK cell numbers, enhanced cytotoxicity, or altered secretory functions may disrupt placental immune balance [74]. Nevertheless, the exact role and mechanisms of dNK cells in SLE-associated abnormal pregnancy outcomes still need to be more thoroughly investigated. Further studies should focus on the dynamic alterations and their direct links to placental dysfunction to uncover the mechanisms of local immune imbalance during pregnancy in SLE.

The application of single-cell RNA sequencing technology has offered new insights into the immune microenvironment in patients with SLE. Lien *et al*. [70] revealed that the distinct immune profile of SLE patients compared with that of healthy controls and RA patients is primarily attributed to the IFN signature characteristic of SLE. In contrast to the pregnancy-associated downregulation of IFN-related genes in healthy controls, SLE patients show marked expansion of IFN-regulated T cells (SC-T3), monocytes (SC-M2), and nonclassical monocytes (SC-M3) [70]. Notably, SC-M3 expansion is partially reversed, whereas SC-M2 gains a more prominent position in the immune cell composition [70]. These findings suggest that altered immune cell composition may contribute to disease activity and pregnancy outcomes in patients with SLE. Relevant studies indicate that SLE patients have increased disease activity at 6 and 12 months after delivery, which aligns with results from the RevNatus registry [75], [76]. These results emphasize the importance of close monitoring and management during the first postpartum year, given the incomplete immune restoration. Personalized immune monitoring and treatment during pregnancy are recommended for SLE patients.

### 3.2 Rheumatoid arthritis

SLE is one of the most common systemic AIDs encountered during pregnancy, whereas RA, another common disease that poses different challenges in pregnancy care, is a complex autoimmune disorder characterized by immune-mediated inflammation of the synovium [77], along with cartilage and bone erosion, primarily impacting multiple joints, particularly small joints in the hands and feet. Pregnant women with RA are especially vulnerable to APOs, including higher rates of low birth weight and stillbirth [78], [79]. Disease control and medication management critically influence pregnancy outcomes [80], [81], [82].

Immune cells, especially CD4^+^ T cells (e.g., Th17 cells), B cells, synovial macrophages, and synovial fibroblasts, are highly accumulated in the local synovium, and participate in chronic inflammation and bone destruction [83], [84]. T cells in RA patients exhibit abnormal activation and differentiation. For example, substantial quantities of proinflammatory cytokines are generated by Th17 cells and CD4^+^CD28^-^ T cells, thereby exacerbating joint inflammation [85]. Furthermore, autoreactive B cells contribute by producing rheumatoid factor and anti-citrullinated protein antibodies (ACPAs), forming immune complexes that deposit in joints, activate complement, and exacerbate inflammation and tissue damage [86]. These alterations in the immune microenvironment collectively promote persistent inflammation and joint damage in RA.

In contrast to SLE, RA displays unique immune changes and a distinct disease course throughout pregnancy. This phenomenon of symptom remission, first observed by Hench in 1938, has since been confirmed by multiple studies [87], [88], [89]. Extensive research indicates that 75%–95% of RA patients undergo remission of disease activity, which is most evident in early pregnancy, peaks in late pregnancy [90], [91], whereas approximately half of patients experience disease flares postpartum [92]. Multiple mechanisms within the immune system help to contribute to the pregnancy-related improvement of RA, whereas the cessation of postpartum immunoregulatory processes is associated with a flare-up of the disease after delivery. Maternal-fetal HLA disparities, immunoglobulin (Ig)G galactosylation, and immunoregulatory mechanisms, along with alterations in innate and adaptive immune cells and their associated cytokines, are pivotal factors in improving RA symptomatology during pregnancy [92], [93], [94]. IgG galactosylation is a pregnancy-associated phenomenon that reduces the pathogenicity of disease-specific autoantibodies. An increase in the galactosylation of anti-cyclic citrullinated peptide IgG during pregnancy is linked to the improvement of RA [95]. In addition, research has shown that patients who test positive for ACPAs experience more limited improvement in RA disease activity [92], [96]. ACPAs represent the most RA-specific autoantibody, and alterations in galactosylation pattern are strongly tied to disease activity [95], [97]. Bondt *et al*. [98] discovered that ACPA-IgG galactosylation was markedly elevated, correlating with enhanced Disease Activity Score in 28 joints using C-reactive protein (DAS28-CRP) levels in RA patients positive for the autoantibody. Meanwhile, galactosylation changes in total IgG did not significantly correlate with disease improvement, suggesting that galactosylation of disease-specific autoantibodies such as ACPA-IgG, is pivotal in the alleviation of RA symptoms [98]. Indeed, galactosylation changes are implicated in the pathogenesis of AIDs. In addition to RA, disorders such as inflammatory bowel disease (IBD), multiple sclerosis (MS), and autoimmune thyroid disease (AITD) also exhibit galactosylation abnormalities [99], [100]. Nevertheless, alterations in galactosylation in these diseases have yet to be reported.

This immune trajectory helps explain why RA often improves during pregnancy but flares after delivery. During the initial phase of pregnancy, paternal antigens (e.g., sperm, TGF-β, prostaglandins, soluble HLA molecules, and other bioactive molecules) and M1 macrophages drive a proinflammatory state to promote implantation [101], [102]. To prevent fetal rejection, FOXP3^+^ Tregs and M2 macrophages are subsequently induced, a Th2-type immune response is adopted, effector T cells are downregulated, and anti-inflammatory cytokines (e.g., IL-10) are produced, leading to an immune tolerance state during pregnancy. As gestation approaches delivery, the immune balance shifts back to a proinflammatory state in preparation for delivery. Within 12 weeks postpartum, CD4^+^ and CD8^+^ T cells exhibit increased activation, along with increased levels of inflammatory mediators. Prolactin activates B cells, promotes the secretion of proinflammatory cytokines, modulates Treg function, and stimulates macrophages to release multiple cytokines [103], [104]. Additionally, recent findings highlight that microchimeric cells may initiate autoimmune responses by presenting as persistent cells or antigens in the maternal system, acting as sources of RA-specific autoantibodies and participating in the activation or aggravation of postpartum autoimmune reactions [105]. This mechanism provides new insights into understanding pregnancy-associated variations in RA disease progression.

RNA sequencing and gene expression analyses provide insights into immune microenvironment changes in pregnant RA patients, particularly regarding IFN-I signaling, B cell function, and neutrophil activity. Studies have shown that women with RA who experience symptom improvement exhibit a marked upregulation of IFN-I-induced genes, whereas those whose symptoms worsen do not show such changes [106], [107], [108]. These findings highlight the role of IFN-I signaling in modulating immune tolerance and inflammatory responses in pregnancy-related RA progression. RNA-seq analysis revealed distinct pre-pregnancy (T0) gene expression profiles between the improvement and deterioration groups. Co-expression network analysis showed that B cell-associated genes, including *CD19*, *CD22*, *CD79A/B*, were enriched in the deterioration group, suggesting that B cells drive adverse immune responses [109]. Conversely, neutrophil-related genes, including folate receptor gamma (*FOLR3*), alanyl aminopeptidase (*ANPEP*), were upregulated in the improvement group, indicating enhanced neutrophil activity [70]. Collectively, these findings emphasize dynamic immunological shifts during pregnancy and the critical roles of B cells and neutrophils in RA pathogenesis.

Despite existing studies providing robust evidence, gaps remain concerning the changes in the local immune microenvironment of RA patients, particularly in the placenta and decidua. Unlike other AIDs, RA often shows a characteristic course of improvement during pregnancy followed by postpartum relapse. This distinctive “pregnancy remission-postpartum relapse” phenomenon has attracted extensive research attention [110], [111]. However, most studies have focused on systemic immune changes, while the specific role of the local immune microenvironment in RA remains unclear. Clarifying these mechanisms will enable the development of more targeted immunoregulatory strategies for RA management.

### 3.3 Antiphospholipid syndrome

Unlike the systemic inflammation driven by joint involvement in RA, the pregnancy risks associated with antiphospholipid syndrome (APS) are mainly attributable to aPLs and blood hypercoagulability, rather than direct inflammatory damage. APS is an autoimmune disorder predominantly characterized by recurrent thrombosis and APOs. Pregnant women with APS face greater risks for both maternal and fetal outcomes [112], [113]. Maternal complications commonly include pregnancy-induced hypertension, PE, and thromboembolic events [114], [115], [116]. Fetal outcomes include significantly higher incidences of miscarriage, preterm birth, intrauterine growth restriction (IUGR), and stillbirth [117]. Research has indicated that the risk of APOs in APS patients can reach approximately 50% [118], and these risks are closely related to the types and titers of aPLs as well as other clinical manifestations (SLE) [117], [119]. The key pathological marker of APS is the presence of aPLs. By binding to phospholipids, aPLs form immune complexes that trigger complement activation, platelet aggregation, and endothelial injury, thus increasing the risk of thrombosis. In addition, aPLs further stimulate inflammatory responses and immune complex formation by activating the complement system, ultimately causing endothelial damage and thrombosis [120], [121]. While aPLs are characteristic of APS, they can also be detected in other AIDs, such as SLE [122], implying that these antibodies play an important part in the immune responses of those ailments as well.

aPLs significantly influence the placenta and the maternal immune system in APS patients. The high expression of β2-glycoprotein I (β2GPI) in the placenta makes it a primary target for aPLs, and its binding can induce endothelial cell phenotypic changes, platelet aggregation, and coagulation dysfunction, with complement system activation acting as a critical mediator in this process [123], [124]. This pathological change reduces trophoblast invasion capacity, resulting in early pregnancy loss; if pregnancy continues, a poorly perfused placenta may lead to complications such as PE and IUGR [125].

As key effector cells of innate immunity, neutrophils exhibit abnormal activation in pregnant women with APS, leading to a significant increase in the release of neutrophil extracellular traps (NETs). These NETs participate in autoimmune pathogenesis and directly impair normal placental development by suppressing trophoblast invasion and migration capabilities [126]. Furthermore, NETs may negatively influence the migratory and tubulogenesis capacities of human umbilical vein endothelial cells, further increasing the likelihood of thrombosis. Moreover, during the adaptive immune response, β2GPI-reactive CD4^+^ T cells accelerate immune dysregulation by activating B cells, leading to enhanced secretion of anti-β2GPI antibodies. aPLs can also bind to their target antigen β2GPI, activating membrane receptors and downstream signaling pathways, which can in turn lead to NK cell activation [127]. In both primary antiphospholipid syndrome (PAPS) and secondary antiphospholipid syndrome (SAPS), research has shown that patients present significantly elevated absolute counts of total T cells and CD4^+^ T cells in peripheral blood, along with a marked Th1/Th2 imbalance manifested by increased Th1 cells, reduced Th2 cells, decreased Tregs, and an increased Th17/Treg ratio [128]. Notably, alterations in T cell subsets in APS patients are strongly correlated with their clinical phenotypes. Patients with thrombotic APS often present elevated proportions of activated CD4^+^HLA-DR^+^ and CD8^+^HLA-DR^+^ T cells, characterized by the upregulation of HLA-DR [129]. Among obstetric APS patients, those without thrombosis present higher proportions of CD4^+^CD45RA CCR7^+^ memory T cells and a higher frequency of activated CD4^+^CD25^+^ subsets [130]. These findings suggest that T cell changes in thrombotic APS are more closely linked to proinflammatory and pro-thrombotic processes, whereas obstetric APS may involve specific regulation of the placental immune microenvironment.

B cells are key contributors to the development of APS. Research has shown that peripheral naive B cells are significantly elevated in both proportion and absolute number, whereas memory B cells are reduced. This abnormal pattern correlates with low complement C4 levels and high titers of IgG anticardiolipin and anti-β2GPI antibodies [130]. Naive B cells potentially facilitate disease progression *via* antigen presentation and autoantibody production. Their increased numbers may reflect defective B cell trafficking, peripheral overactivation, or abnormal differentiation into plasma cells, causing memory B cell loss. These findings indicate that B cell subset imbalance is a critical immunologic hallmark of thrombosis in APS patients and a potential factor contributing to poor pregnancy outcomes in affected patients.

aPLs contribute to pregnancy complications through multiple pathways, and their pathogenic effects extend beyond thrombosis. For example, anti-β2GPI antibodies bind to β2GPI on endothelial cells, triggering the coagulation cascade, inducing tissue factor expression, and promoting platelet activation and aggregation, thereby driving microthrombi formation in placental and umbilical vessels, impairing placental perfusion, and ultimately increasing the risk of adverse pregnancy outcomes [131]. Moreover, aPLs can specifically disrupt the anticoagulant annexin V barrier on trophoblast surfaces [132], [133], increasing the susceptibility of placental cells to abnormal apoptosis and differentiation disorders [134]. Concurrently, aPLs induce local inflammation *via* complement activation and promotes proinflammatory cytokine secretion, contributing to a prothrombotic and proinflammatory microenvironment. Nevertheless, the precise molecular mechanisms through which aPLs contribute to pregnancy complications have yet to be fully elucidated. Further studies are needed to clarify these unresolved mechanisms, which will provide theoretical support for the development of novel targeted therapies to inhibit the pathogenicity of aPLs.

### 3.4 Sjögren’s syndrome

Sjögren’s syndrome (SS) is a chronic immune-mediated disease that predominantly targets the salivary and lacrimal glands, resulting in prominent dryness of the mouth, eyes, and skin, and is often accompanied by fatigue, joint pain, and neuropsychiatric symptoms. Investigations into reproductive outcomes in primary Sjögren’s syndrome (pSS) patients have yielded mixed and somewhat controversial findings. A multicenter, prospective cohort study in France indicated that, relative to people in general, women with pSS generally have a better prognosis during spontaneous pregnancy, with no substantial elevation in the overall risk of APOs [135]. However, for pSS patients undergoing in vitro fertilization, a multicenter retrospective cohort study found that they had poorer fertility and clinical pregnancy status, especially those characterized by oocyte and embryo developmental abnormalities [136]. This discrepancy suggests that pSS may impact pregnancy outcomes differently under natural conception and assisted reproductive technology, indicating potential mechanistic differences that warrant further investigation. Beyond maternal pregnancy outcomes, pSS also carries fetal and neonatal risks mediated by autoantibodies. Mothers carrying anti-Sjögren’s syndrome-related antigen A (SSA/Ro) and anti-Sjögren’s syndrome-related antigen B (SSB/La) autoantibodies are at increased risk of neonatal lupus-related cardiac complications, with congenital heart block (CHB) being the most severe complication [137].

The main characteristic of SS is immune system dysregulation, particularly with respect to aberrant T and B cell activation. In SS patients, T cell infiltration, with a predominance of CD4^+^ subsets, is markedly evident within the salivary glands [138], [139]. Research indicates that multiple T cell subtypes, including Th1, Th2, Th17, and Tregs, are fundamentally involved in the onset and progression of SS [140], [141]. Th1 cell activation enhances the secretion of inflammatory mediators. Moreover, Th2 cells spur excessive B-cell activation by releasing cytokines, which in turn boost the generation of autoantibodies (e.g., anti-SSA/Ro and anti-SSB/La), exacerbating the immune response in SS [142], [143].

Current research on the changes in the immune microenvironment of SS during pregnancy is still quite limited. Autoantibodies, especially anti-SSA and anti-SSB, are present in most SS patients and can traverse the placental barrier, potentially triggering immune complications in the fetus. Upon fetal exposure to maternal Ro/La antibodies, these antibodies can be actively transferred to the fetal circulation *via* trophoblastic Fcγ receptors. These antibodies recognize SSA/Ro-SSB/La antigens exposed on fetal cardiomyocytes due to physiological apoptosis during cardiac development, triggering proinflammatory phagocytic responses that lead to irreversible fibrotic injury of the conduction system [144]. The risk of neonatal lupus-related cardiac complications is elevated, with CHB representing the most severe complication [145]. Ivanchenko *et al.* [146] noted changes in B-cell subpopulations, relatively fewer T cells, and lower NK cell frequencies in Ro/La^+^ mothers, which aligns with the immunological features observed in SS. Notably, in newborns exposed to Ro/La antibodies, the frequency of CD56^dim^CD16^high^ NK cells was increased, suggesting that their NK cells may be activated and exhibit cytotoxic activity. IFN-α is considered critical to this pathway because it drives NK cell proliferation. *Via* antibody-dependent cellular cytotoxicity (ADCC), NK cells bind to Ro/La antibodies through CD16, resulting in myocardial cell injury and culminating in CHB.

The interactions between Ro/La antibodies, IFN, and cytotoxic cells may also constitute one of the core mechanisms underlying the pathogenesis of this disease. These immune mechanisms not only influence the maternal immune microenvironment but also affect the immune response of newborns through the placenta [145]. Thus, investigating the function of Ro/La antibodies reveals the intricate alterations in immune cell groups during pregnancy, offering new insights into the mechanisms underlying SS-related pregnancy complications and potentially providing theoretical support for future diagnostic and treatment strategies.

### 3.5 Multiple sclerosis

MS, a chronic AID of the central nervous system, presents a distinct pregnancy-associated immune profile. MS is characterized by immune-mediated demyelination and axonal injury, driven by CD4^+^ T cells (Th1, Th17), glial cells, B cells, dendritic cells, and macrophages [147], [148], [149]. Th1 and Th17 cells promote central nervous system inflammation through cytokine secretion, whereas B cells exacerbate demyelination *via* antibody production and complement activation [150], [151], [152]. Dendritic cells and macrophages enhance immune cell activation and migration, and glial cell dysfunction under immune dysregulation may contribute to neuronal injury [153], [154]. This dysregulated immune microenvironment sustains neural damage and disease progression.

During pregnancy, the immune microenvironment of MS experiences intricate and important alterations, significantly affecting disease activity and clinical presentation. Hormonal fluctuations, particularly in estrogen, progesterone, and testosterone, contribute to a reduced relapse rate [155], [156]. Estradiol limits encephalitogenic T cell activation and central nervous system infiltration *via* its α receptor, exerting neuroprotective effects, whereas estriol enhances Treg expansion and suppresses Th17 differentiation, further alleviating disease activity [157], [158]. However, these immunomodulatory effects are often disrupted after delivery, and many MS patients experience a transient worsening of their condition postpartum [159]. This phenomenon suggests that the immune tolerance and regulatory effects observed are transient and that postpartum immune tolerance may revert to a heightened inflammatory state. In addition to hormonal influences, IFN may also regulate disease activity in MS patients. While IFN-I signatures are linked to disease activity in SLE, IFN biology in MS appears more nuanced: exogenous IFN-I has well-established therapeutic efficacy in MS, whereas the contribution of endogenous IFN signaling to pregnancy-related remission and postpartum relapse remains unclear. Notably, an increased IL-10/IFN-γ ratio in late pregnancy may promote immune tolerance, potentially representing another mechanism underlying MS remission [160]. Furthermore, a similar “pregnancy remission-postpartum relapse” pattern observed in RA patients suggests that pregnancy-related immune regulation may involve shared pathways across different AIDs [108], [161]. A comprehensive exploration of the involvement of IFN in pregnancy-associated AIDs could advance the understanding of MS remission mechanisms and potentially offer new therapeutic targets for preventing postpartum relapse.

In patients with MS, autoreactive T cells activated in the periphery demonstrate significant abnormalities, most notably those of the CD4^+^ subset [162], [163]. Among untreated, nonpregnant individuals with MS, the peripheral blood shows a considerably increased level of CD4^+^ T cells compared with that of healthy controls [164], which is in line with the characterization of MS as a CD4^+^ T cell-mediated immunopathology. Notably, during pregnancy, individuals with MS continue to display an elevated proportion of CD4^+^ T cells, with no significant changes observed compared to the nonpregnant period. This may obscure the alterations in immune cell subsets involved in immune regulation [164]. For instance, MS patients exhibit a shift from Th1-type to Th2-type immune responses, which reverts back to Th1-type responses after delivery [160], [165]. This shift may be a contributing factor to the exacerbation of MS symptoms postpartum. In addition to Th cells, the function of Tregs in MS remains controversial [166], [167], [168]. Many investigations have suggested that in healthy women, Treg counts drop during gestation and return to pre-pregnancy levels postpartum. However, in women with MS, the Treg frequency remains constant throughout pregnancy, without a significant postpartum rebound or decline relative to the the Treg frequency in nonpregnant women [169]. These observations conflict with some reports indicating that Treg counts in pregnant MS patients may either increase or decrease [170]. Importantly, many studies rely on FOXP3 as a Treg marker, and despite FOXP3 being critical for Treg development and function, it can also be upregulated in newly activated conventional T cells, leading to potential misinterpretation when identifying and characterizing Tregs [171], [172], [173].

NK cells also contribute to immune modulation in pregnant MS patients. The peripheral NK cell profiles shift, with increased CD56^bright^ and decreased CD56^dim^ subsets, mirroring the changes observed in healthy pregnancies [174]. These shifts typically reverse postpartum, indicating that pregnancy-induced NK cell regulation is transient. However, in MS patients, CD56^bright^ NK cells have a reduced capacity to suppress autologous CD4^+^ T cell proliferation compared with that in healthy controls [169], suggesting impaired regulatory function, which may limit clinical improvement during pregnancy.

Locally, similar changes occur in the decidua. CD56^bright^ NK cells, although enriched in the decidua relative to those in peripheral blood, are lower in pregnant MS patients than in healthy pregnant women, implying compromised immunoregulation [169]. Decidual macrophages predominantly adopt an M2 phenotype, derived from CD14^+^CD163^+^ monocytes, contributing to immune tolerance, and resembling anti-inflammatory macrophages found in MS [175], [176]. In addition, placental extracellular vesicles and decidual mesenchymal stem cells play vital roles in immunoregulation. Extracellular vesicles maintain immune tolerance by modulating the proliferation and differentiation of immune cells, whereas decidual mesenchymal stem cells inhibit Th1 and Th17 cells and enhance Treg activity, further supporting immune tolerance during gestation [177], [178].

In recent years, emerging technologies have offered pivotal insights into the immune microenvironment during pregnancy in MS. Epigenomic and transcriptomic studies have shown that T cells in MS patients display distinctive epigenetic and transcriptional alterations, correlating with disease remission and reversal after delivery [179], [180]. Moreover, proteomic analysis further revealed a characteristic profile in which immunomodulatory proteins, such as programmed death ligand 1 (PD-L1) and leukemia inhibitory factor receptor (LIF-R), were upregulated, whereas inflammatory mediators, including C-C motif chemokine ligand 8 (CCL8) and C-X-C motif chemokine ligand 5 (CXCL5), were downregulated in gestation [181], offering critical insights for understanding pregnancy-related immune regulation and identifying novel biomarkers.

### 3.6 Other autoimmune diseases

The immunomodulatory impact of pregnancy is not limited to prototypical autoimmune rheumatic diseases, it can also be observed in other autoimmune conditions, such as IBD and AITD. Despite having different pathogenic mechanisms, IBD and AITD both exhibit unique immune regulation patterns during pregnancy.

IBD is a chronic disorder characterized by intestinal inflammation, including Crohn’s disease (CD) and ulcerative colitis (UC). CD is mainly characterized by transmural inflammation mediated by Th1/Th17 immune responses, whereas UC predominantly involves a Th2/Th17 mixed immune response, with lesions typically confined to the colonic mucosa [182]. The risk of disease activity also differs between IBD subtypes, reaching up to 70% in UC compared with 54% in CD, although this difference is not statistically significant [183]. Biomarker profiles also reflect subtype-specific patterns. IL-6 levels in early pregnancy may predict CD activity, whereas IL-22 levels correlate with UC progression [184]. Moreover, sex hormones and immunoregulatory factors significantly influence the immune microenvironment in patients with IBD. In Th1-type experimental colitis, exogenous estrogen can reduce inflammation by downregulating proinflammatory cytokines and mast cell proteases *via* an estrogen receptor (ER)-dependent mechanism [185]. Placental trophoblast-derived pregnancy-specific glycoproteins (PSGs) and TGF-β contribute to anti-inflammatory regulation by downregulating Th1-mediated inflammation and enhancing Treg populations. Nonetheless, active IBD is significantly linked to poor pregnancy outcomes [186], indicating that physiological immune adaptations may inadequately counterbalance the pathological processes of IBD.

AITD displays unique immunoregulatory features during pregnancy, mainly encompassing two prevalent subtypes, Hashimoto’s thyroiditis (HT) and Graves’ disease. The immune system of AITD patients undergoes changes that may impact maternal health, pregnancy outcomes, and fetal development. AITD is closely associated with APOs, including placental abruption, spontaneous miscarriage, preterm birth, pregnancy-induced hypertension, and IUGR [187]. Notably, elevated levels of thyroid peroxidase antibodies and thyroglobulin antibodies are strongly linked to a greater risk of gestational hypertension and are negatively correlated with neonatal birth weight [188]. Therefore, the immune microenvironment of AITD patients not only affects maternal disease activity but also influences pregnancy outcomes and fetal development through various mechanisms. Mechanistically, AITD is characterized by coordinated dysregulation of innate and adaptive immunity. Dendritic cells and macrophages initiate T cell activation through the presentation of thyroid antigens, orchestrating the immune responses of Th1, Th17, and Treg subsets. Macrophage infiltration within the thyroid can induce localized chronic inflammation in HT patients, thereby intensifying thyroid dysfunction [189]. Furthermore, macrophages are capable of producing proinflammatory cytokines, which amplify inflammatory reactions and further facilitate the recruitment and activation of immune cells, thus promoting immune-mediated injury [189], [190]. In patients with Graves’ disease, neutrophils exacerbate thyroid damage and inflammation by releasing NETs and secreting proinflammatory factors [191]. Although the immune system generally shifts towards an anti-inflammatory state, Th1 and Th17 activation may persist in patients with AITD. This persistence may be related to fluctuations in pregnancy-associated hormones and contribute to disease relapse or worsening during pregnancy and the postpartum period [192]. Moreover, the function of NK cells may also change. Although studies indicate that their numbers may decrease, this does not imply a loss of their role in immune regulation. Various subsets of NK cells exert immunoregulatory functions, potentially having a significant impact on immune microenvironment regulation in AITD [193], [194].

## 4 Clinical management and treatment strategies

The clinical management of AIDs during pregnancy should consider both maternal health and fetal safety. Personalized treatment approaches are essential for effectively managing the disease while minimizing the potential adverse effects of medications on the fetus. The primary goal is to achieve a balance between controlling the disease and ensuring the safety of both the mother and the fetus. Consequently, pre-pregnancy assessments, continuous monitoring and diligent follow-up are essential elements of the management plan. Table 1 summarizes the clinical management strategies for common AIDs during pregnancy, including preconception evaluation and pregnancy management [64], [184], [185], [195], [196], [197], [198], [199], [200].

**Table 1:** Clinical management strategies for common autoimmune diseases (AIDs) during pregnancy.

**Disease type**	**Pre-pregnancy assessment and preparation**	**Monitoring and follow-up during pregnancy**	**Precautions during pregnancy**	**References**
SLE	Conduct pre-pregnancy risk stratification to ensure the disease has been in remission for ≥6 months, and adjust medications as needed (for example, discontinue cyclophosphamide); All SLE patients should continue taking HCQ during pregnancy; For individuals with no plan to become pregnant, provide contraceptive information	Conduct regular multidisciplinary follow-up to evaluate lupus disease activity and potential complications; For pregnant women who test positive for anti-Ro/SSA and/or anti-La/SSB antibodies but have no history of infants with CHB or NLE, it is recommended to perform weekly fetal echocardiography starting from 16*–*18 weeks until 26 weeks	SLE patients during pregnancy should be closely monitored for both fetal and maternal well-being, and the use of medications harmful to the fetus should be avoided	[184], [185], [195]
RA	Perform pre-pregnancy antibody screening (anti-Ro/SSA, anti-La/SSB, and aPL antibodies); Evaluate pre-pregnancy factors such as disease activity, use of teratogenic medications, and obstetric history; Discuss pregnancy plans before conception and strive to achieve clinical remission through pharmacological control of the disease. It is recommended that disease remain stable for at least six months prior to pregnancy; Provide contraceptive information to those who are not planning to conceive	Monitor joint function and regularly assess disease activity; Evaluate medication safety and avoid drugs harmful to the fetus; Monitor weight gain and blood pressure levels throughout pregnancy; For pregnant women positive for anti-Ro/SSA and/or anti-La/SSB antibodies but without a history of infants with CHB or NLE, weekly fetal echocardiography is recommended starting at weeks 16*–*18 and continuing through week 26	Avoid medications that may affect fetal development, such as biologics, prior to delivery. During pregnancy, give priority to disease control and fetal safety	[64]
APS	Assess early risk factors before pregnancy (such as AIDs, history of miscarriage, thrombosis, aPL antibodies, and hypocomplementemia); Discuss pregnancy plans before conception and aim to achieve clinical remission of the disease through pharmacological management. It is recommended to maintain stable disease activity for at least six months prior to pregnancy; For individuals not planning to conceive, provide information on contraception	From pregnancy through 6*–*12 weeks postpartum, prophylactic doses of LMWH and low-dose NSAIDs should be administered; for vascular APS patients, therapeutic doses of heparin plus low-dose ASA are indicated during pregnancy. Women positive for aPL antibodies but without prior APS history should receive low-dose ASA to reduce the risk of preeclampsia; During anticoagulation, monitor coagulation parameters, platelet counts, and liver/kidney function; In the mid to late stages of pregnancy, monitor for diseases associated with poor placental function; For pregnancies involving APS, it is essential to monitor maternal and fetal health continuously and decide on the best timing for delivery	Pregnant APS patients face elevated risks of placental dysfunction and miscarriage, necessitating close surveillance for thrombosis and fetal well-being	[199]
pSS	Discuss pregnancy plans before conception and aim to achieve clinical remission of the disease through pharmacological management. It is recommended to maintain stable disease activity for at least six months before pregnancy; Perform a preconception assessment including factors like aPL antibodies, anti-SSA/Ro antibody profiles, disease activity, use of teratogenic drugs, history of miscarriages, and thrombosis; Recommend that pregnant women adjust to pregnancy-compatible medications and supplement specific vitamins	All pregnant women positive for anti-SSA/Ro antibodies should take HCQ; For pregnant women who test positive for anti-Ro/SSA and/or anti-La/SSB antibodies but have no history of infants with CHB or NLE, it is recommended to perform weekly fetal echocardiography starting from weeks 16*–*18 until week 26; Perform routine prenatal check-ups, which should include monitoring blood pressure, assessing fetal growth, and evaluating cardiac function	For individuals testing positive for anti-Ro/SSA and anti-La/SSB antibodies, conduct fetal electrocardiogram monitoring during early pregnancy, with management jointly handled by obstetricians and pediatric cardiology experts; Regularly monitor blood pressure to promptly detect and manage hypertension-related complications; Promote and support breastfeeding	[200]
MS	Females diagnosed with MS should give precedence to annual preconception consultations, particularly if they are undergoing treatment or intend to begin therapy; Prior to pregnancy, engage in discussions about conception plans and endeavor to manage the disease with medications to achieve clinical remission. It is advised to ensure disease stability for a minimum of 6 months before attempting pregnancy; For patients with high disease activity, it is generally recommended to postpone pregnancy; Perform an extensive preconception assessment of the patient's general health to confirm the absence of significant complications and ensure that folic acid supplementation is initiated prior to pregnancy, thereby minimizing the risk of neural tube defects in the fetus	During pregnancy, closely track any changes in patients’ symptoms. In the event of an acute flare-up, early assessment and intervention are essential; Avoid using medications that may pose potential risks to the fetus during pregnancy. If treatment is necessary, consider using medications that are relatively safer for use during pregnancy; Pregnant MS patients might face an increased number of obstetric complications, requiring diligent monitoring and timely interventions from obstetric care providers	Pregnant patients with MS require frequent follow-ups, including disease monitoring and fetal surveillance; Postpartum depression poses a potential risk for individuals with MS, underscoring the importance of psychological evaluations and supportive therapies; It is recommended that pregnant individuals with MS engage in breastfeeding after childbirth and suitably modify disease-modifying treatments and other therapeutic interventions	[196], [197], [198]

SLE. Systemic lupus erythematosus; HCQ. Hydroxychloroquine; CHB. Congenital heart block; NLE. Neonatal lupus erythematosus; RA. Rheumatoid arthritis; SSA/Ro. Sjögren’s syndrome-related antigen A; SSB/La. Sjögren’s syndrome-related antigen B; aPL. Antiphospholipid; APS. Antiphospholipid syndrome; AID. Autoimmune disease; LMWH. Low-molecular-weight heparin; NSAID. Non-steroidal anti-inflammatory drug; ASA. Aminosalicylic acid; pSS. Primary Sjögren’s syndrome; MS. Multiple sclerosis

### 4.1 Pre-pregnancy evaluation and preparation

A comprehensive pre-pregnancy assessment is crucial to ensure maternal and fetal safety. For patients with AIDs complicated by pregnancy, a joint evaluation by rheumatology and obstetrics specialists should be conducted before conception to ensure that the disease is in a stable or remission phase. Studies have shown that after receiving counseling and treatment before pregnancy, the majority of patients achieve disease remission in late pregnancy, and pregnancy outcomes are significantly improved compared with those of pregnant women who do not receive preconception counseling and disease treatment [201], [202], [203]. According to the latest guidelines from the European League Against Rheumatism (EULAR), it is necessary to confirm that patients exhibit no active organ involvement before pregnancy, modify medications that are teratogenic, and ensure a safe pregnancy only when the disease is well-controlled [204]. For example, the American College of Rheumatology (ACR) advises that all women diagnosed with SLE should take HCQ during pregnancy whenever feasible [204]. Research indicates that the combination of HCQ and low-dose aspirin significantly enhances pregnancy outcomes in SLE patients by regulating the Th1/Th2 cytokine equilibrium [205]. If the patient is already taking HCQ, it is recommended to continue its use; if not, HCQ should be initiated, provided that no contraindications are evident. Moreover, the Global Antiphospholipid Syndrome Score (GAPSS) functions as a clinical tool capable of accurately predicting the response of aPL antibody-positive patients to combined aspirin and low-molecular-weight heparin (LMWH) therapy [206]. Additionally, performing both medical and investigative assessments to gauge overall disease progression and maternal-fetal risks, such as assessing disease activity (e.g., complement levels), screening for anti-Ro/SSA and anti-La/SSB antibodies, and testing for aPL antibodies, is vital. These assessments facilitate the creation of personalized treatment regimens and the adjustment of pregnancy management strategies as needed. For patients deemed high-risk, it is advisable to postpone pregnancy until the disease is sufficiently managed, usually necessitating a minimum of 6 months of pregnancy management strategies to mitigate potential risks during gestation.

### 4.2 Prenatal monitoring and follow-up

Throughout pregnancy, rigorous monitoring is needed to detect and manage shifts in disease activity and fetal development in a timely manner. Periodic clinical assessments, alongside lab work (e.g., hepatic/renal function and antibody titers), can aid in gauging disease control. For example, because active disease can affect both maternal health and pregnancy outcomes, the ACR strongly recommends at least one assessment of SLE disease activity during pregnancy, including a review of clinical history, physical examination, and laboratory testing [199].

For pregnant women with anti-Ro/SSA and/or anti-La/SSB antibodies but without a history of infants with CHB or neonatal lupus erythematosus (NLE), it is recommended to perform serial fetal echocardiography from 16 to 18 weeks, continuing through 26 weeks [199]. For women with a history of having CHB or other NLE infants, weekly fetal echocardiography should be performed from 16 to 26 weeks to facilitate early detection of potential fetal cardiac abnormalities and ensure timely intervention [199], [204]. This form of surveillance helps detect possible pregnancy-related complications early and secures optimal management strategies throughout pregnancy. Notably, recent studies have confirmed that elevated peripheral blood IFN-α protein levels in pregnant SLE patients are independently associated with low birth weight in neonates [207], [208]. However, the prognostic utility of IFN signaling for routine risk stratification requires confirmation in large-scale prospective multicenter studies.

### 4.3 Special case management

Throughout pregnancy, certain patients with autoimmune rheumatic disorders may undergo notable fluctuations in disease activity or develop pregnancy-associated complications, requiring prompt and effective treatment adjustments to safeguard both the mother and fetus. For example, pregnant RA patients can experience variations in disease status, presenting with either worsening of, or alleviation of, their joint inflammation. To reduce risks to the fetus, individuals with RA are advised to cease methotrexate (MTX) prior to conception and use medications deemed safe for fetal health during pregnancy, such as sulfasalazine or HCQ, with dosage modifications made to sustain disease control [64]. When RA patients develop severe pregnancy complications, such as acute arthritis or organ involvement, a multidisciplinary team composed of rheumatologists, obstetricians, and other relevant specialists should collaborate to devise an individualized treatment plan, potentially including the use of fetal-safe corticosteroids and biologics [64]. Moreover, high-risk pregnancy conditions, including PE and placental abruption, require careful monitoring by obstetricians, and urgent delivery should be considered if circumstances warrant. In critical cases where disease flares are unmanageable or pose a grave risk to the mother’s survival, terminating the pregnancy prematurely becomes an essential safeguard [209].

### 4.4 Medication selection and safety considerations

In the clinical management of AIDs, medication selection requires careful consideration of both effective disease control and the safety of the fetus. According to the FDA’s Pregnancy and Lactation Labeling Rule (PLLR) [210], drug labels must provide detailed information on medication use during pregnancy and lactation to assist healthcare professionals and patients in better assessing the risks and benefits of the drug. Based on current research and clinical data, relatively safe medications include corticosteroids, sulfasalazine, and LMWH, all of which are frequently utilized to manage disease activity and mitigate the risk of APOs [210]. Medications requiring caution, such as azathioprine, nonsteroidal anti-inflammatory drugs, and biologic agents, call for close monitoring of maternal and fetal health when used. Prohibited medications include chlorambucil, cyclophosphamide, leflunomide, and MTX as they can lead to severe teratogenic effects [210]. Through careful selection of therapies and close surveillance, it is possible to effectively balance disease management with maternal and fetal safety, thereby offering the safest treatment approach for pregnant patients. Table 2 summarizes the pharmacokinetic characteristics of common autoimmune medications, along with their effects on the immune microenvironment, therapeutic efficacy, and safety considerations [64], [117], [207], [211], [212], [213], [214], [215], [216], [217], [218], [219], [220], [221], [222], [223], [224], [225], [226], [227], [228], [229], [230], [231], [232], [233], [234], [235], [236], [237], [238], [239], [240], [241], [242], [243], [244], [245], [246], [247], [248], [249], [250], [251], [252], [253], [254], [255], [256], [257], [258]

**Table 2:** Common drugs for autoimmune sexually transmitted diseases during pregnancy and their safety evaluation.

**Drug category**	**Drug name**	**Pregnancy indications**	**Pharmacokinetics and effects on the immune microenvironment**	**Efficacy and biological safety**	**References**
Safe medications	Corticosteroids	SLE, RA, APS	Dose-dependent characteristics (oral clearance, distribution volume, and free drug fraction all increase with higher doses or concentrations); Inhibits Th1-mediated proinflammatory factors and enhances Th2-driven anti-inflammatory cytokine production, facilitating a shift from cellular to humoral immunity and enabling selective immunomodulation rather than generalized immunosuppression	The minimum effective dose should be maintained during pregnancy; Long-term high-dose use may increase the risk of preterm birth and low birth weight in infants	[211], [212], [213], [214], [215], [216]
	HCQ	SLE, RA, SS, autoimmune, congenital heart conduction block	Pregnancy markedly changes the pharmacokinetics of HCQ, showing faster clearance, expanded distribution volume, shortened half-life, and decreased blood drug concentration; By altering the pH of cellular lysosomes, the antigen-presenting function of macrophages and the secretion of IL-1 are weakened. This pH modification also reduces lymphocyte activation; Capable of disrupting antiphospholipid immune complexes and re-establishing annexin A5 binding to the phospholipid bilayer	Lowering disease activity, the incidence of preterm birth, IUGR, and the risk of preeclampsia in pregnant SLE patients; Treatment with hydroxychloroquine during pregnancy does not elevate the risk of fetal structural malformations or other adverse outcomes; If patients report changes in vision, visual fields, color perception, etc., during medication use, they should undergo prompt ophthalmologic evaluation. Long-term medication users are advised to have regular eye examinations	[220], [221], [222], [223], [224], [225], [226], [227]
	Calcineurin inhibitors (cyclosporine A, tacrolimus)	SLE, SS, refractory RA	Tacrolimus crosses the placenta, with fetal blood drug concentrations reaching about 71% of those in the mother; Inhibit T cell nuclear factor, thereby inhibiting IL-2 synthesis and release, and suppressing and altering T cell proliferation and differentiation	Tacrolimus is an effective adjunctive or alternative therapy to corticosteroids for managing lupus nephritis flares or maintaining stable disease during pregnancy; During use of cyclosporine and tacrolimus, monitor blood pressure, kidney function, and blood potassium levels. Be mindful of drug interactions and monitor blood drug concentrations if necessary	[228], [229], [230], [231]
	LMWH	Primary and secondary APS	Pregnancy markedly affects LMWH pharmacokinetics, with progressively elevated clearance and increased distribution volume, resulting in decreased anti-Xa activity; By enhancing ATIII’s inhibitory effects on factor Xa and thrombin, the formation of thrombosis is effectively prevented, thereby reducing the occurrence of thrombotic-related complications	Combined use of heparin and aspirin increases live birth rates in APS patients; During pregnancy, it is often necessary to use LMWH or combine it with low-dose aspirin, selecting prophylactic or therapeutic doses based on the patient’s condition	[117], [217], [218], [219]
	NSAID	RA, spinal arthritis, APS, SLE	Aspirin pharmacokinetics during pregnancy are influenced by obesity/BMI, leading to reduced plasma concentrations, while its pharmacodynamics demonstrate a direct link between drug levels and platelet inhibition; Total metabolite concentrations of aspirin decrease during pregnancy, while its clearance increases; Decreasing prostaglandin production modulates immune cell activity and cytokine secretion, potentially indirectly inhibiting the proliferation of Th17 cells and the generation of PGE2. This further impacts macrophage function and reduces the secretion of proinflammatory cytokines (like TNF-α and IL-1β), thus mitigating autoimmune reactions	During the mid-pregnancy period, NSAIDs are relatively safe to use, with non-selective COX inhibitors being the first choice. The use of NSAIDs in late pregnancy significantly increases the risk of fetal ductus arteriosus premature closure and should be avoided; It is essential to use NSAIDs during pregnancy, utilizing the minimal effective dose and limiting the duration of use as much as possible	[232], [233], [234], [235]
	Sulfasalazine	RA and spondylarthritis with peripheral arthritis	Following sulfasalazine use during pregnancy, its metabolites can cross the placenta, though fetal exposure to 5-aminosalicylic acid remains minimal; only trace levels of 5-ASA are found in breast milk; In the intestines, it is metabolized into 5-ASA and sulfasalazine. The former inhibits prostaglandins and neutralizes proinflammatory oxygen radicals released by phagocytes. After 12 weeks of taking this medication, activated lymphocytes in peripheral blood decrease in arthritis patients	Sulfasalazine does not increase the risk of fetal malformations or adverse pregnancy outcomes; Pregnant women taking this drug should supplement with folic acid to lower the risks of fetal cleft lip, cardiovascular defects, and urethral malformations; For breastfeeding mothers taking sulfasalazine, normal breastfeeding is safe for healthy full-term infants. However, caution is required when breastfeeding preterm infants, those with glucose-6-phosphate dehydrogenase deficiency, and infants with hyperbilirubinemia; High-dose sulfasalazine (3 g/d) during breastfeeding may cause hemorrhagic diarrhea in infants, breastfeeding or medication should be discontinued if this occurs	[207], [236], [237], [238], [239], [240]
Selective use of safe medications	Azathioprine (AZA)	SLE, SS, etc.	Systematic changes in 6-thioguanine nucleotide (6-TGN) concentrations, a metabolite of AZA, occur during pregnancy and the perinatal period, while concentrations of 6-methylmercaptopurine nucleotide (6-MMPN) are higher during pregnancy; Neonates are only transiently exposed to low concentrations of thiopurine metabolites, which are cleared within 6 weeks without causing anemia; Interfere with the synthesis of adenine and guanine nucleotides, thereby inhibiting the synthesis and growth of activated lymphocytes	The use of AZA during pregnancy is considered safe; Closely monitor complete blood counts to early detect potential bone marrow suppression	[241], [242], [243]
	Tumor necrosis factor inhibitors (TNFi)	RA and spondylarthritis	Certolizumab pegol, lacking an Fc fragment, shows minimal placental transfer and can be continued throughout pregnancy. Other TNFi (e.g., etanercept, infliximab) contain the IgG1 Fc region and cross the placenta increasingly in late pregnancy; thus, discontinuation before the third trimester is recommended; Suppresses TNF-α activity, decreases proinflammatory cytokine secretion, alleviates inflammation, and mitigates damage to cartilage and bone; Limits antioxidant effects, inhibits activation of T cells and macrophages, suppresses necrosis factor production, and reduces local tissue infiltration	TNFi are effective in controlling disease activity in patients with immune-mediated inflammatory diseases, and are relatively safe for use during pregnancy, though attention should be paid to their potential side effects	[244], [245], [246], [247], [248], [249]
	IVIG	Inflammatory myopathies, catastrophic APS, SLE without secondary immunocytopenias	IVIG exposure levels remain stable during pre-pregnancy, early pregnancy, and mid-pregnancy, with a dose-dependent profile; IVIG has roles in modulating lymphocyte immune function, suppressing B cell and antibody activity, blocking Fc receptors, inhibiting complement function, and reducing the activity of NK cells. Moreover, the microbe-antigen-specific binding characteristics of the immunoglobulin IgG-F(ab’)2 fragment can offer passive immunity to the organism	IVIG treatment can result in favorable pregnancy outcomes, yet fetal health status requires close monitoring throughout therapy, particularly in late gestation	[250], [251]
Avoidance of drug use	MTX	RA, psoriatic arthritis and other forms of inflammatory arthritis	MTX inhibits dihydrofolate reductase, reducing the synthesis of tetrahydrofolate, thereby inhibiting the synthesis of pyrimidines and purines, and suppressing immune cell proliferation; Methotrexate polyglutamates inhibit 5-aminoimidazole-4-carboxamide ribonucleotide (AICAR) transformylase, leading to AICAR accumulation and promoting adenosine release, which suppresses inflammation by binding to cell surface receptors	Discontinue medication at least 3 months before pregnancy. Women who have received MTX treatment within 3 months prior to conception should supplement with folic acid before pregnancy and throughout the entire pregnancy. MTX is contraindicated during breastfeeding	[252], [253], [254]
	Cyclophosphamide	SLE, RA, systemic necrotizing vasculitis, progressive systemic sclerosis	The metabolites of cyclophosphamide undergo addition reactions with DNA bases, leading to DNA strand breaks or cross-links, which inhibit DNA replication and transcription, ultimately suppressing cell proliferation and division, producing cytotoxic effects; Suppresses the proliferation of immune cells, especially B and T cells, reduces inflammatory responses, and effectively inhibits abnormal immune reaction	Medication should be discontinued for 6 months prior to conception	[255]
	Leflunomide	Refractory lupus nephritis	By inhibiting DHODH, it disrupts the synthesis of pyrimidine nucleotides, thereby inhibiting DNA and RNA synthesis, especially affecting immune cells that are rapidly proliferating	Discontinue the medication for 2 years before planning pregnancy (if using cholestyramine or other agents to eliminate leflunomide from the enterohepatic circulation, it is recommended to discontinue the medication for 3—6 months)	[64], [256]
	MMF	Moderate to severe lupus nephritis, Rheumatic disease-associated interstitial lung disease, etc.	Inhibition of adenosine deaminase disrupts the synthesis of purine nucleotides, especially impacting the proliferation of B and T cells	Discontinuation of medication is required at least 6 weeks before planning pregnancy	[64], [257]
	Tripterygium wilfordii Hook. f.	RA, Behçet’s syndrome triad, autoimmune hepatitis	By inhibiting T cell proliferation and reducing cytokine release, it regulates abnormal immune responses	Discontinuation of medication is required at least 6 months before planning pregnancy	[64], [258]

SLE. Systemic lupus erythematosus; RA. Rheumatoid arthritis; APS. Antiphospholipid syndrome; HCQ. Hydroxychloroquine; SS. Sjögren’s syndrome; IUGR. Intrauterine growth restriction; LMWH. Low-molecular-weight heparin; NSAID. Non-steroidal anti-inflammatory drug; anti-Xa. Anti-factor Xa; ATIII. Antithrombin III; ASA. Acetylsalicylic acid; BMI. Body mass index; Th17. T helper 17; PGE2. Prostaglandin E2; TNF-α. Tumor necrosis factor-α; IL-1β. Interleukin-1β; COX. Cyclooxygenase; AZA. Azathioprine; TNFi. Tumor necrosis factor inhibitor; 6-TGN. 6-thioguanine nucleotide; 6-MMPN. 6-methylmercaptopurine nucleotide; IVIG. Intravenous immunoglobulin; MTX. Methotrexate; AICAR. 5-aminoimidazole-4-carboxamide ribonucleotide; DNA. Deoxyribonucleic acid; RNA. Ribonucleic acid; DHODH. Dihydroorotate dehydrogenase; MMF. Mycophenolate mofetil

These commonly used immunosuppressive medications improve pregnancy outcomes by modulating dysregulated immune microenvironment in patients with AID. For example, corticosteroids promote a shift from proinflammatory Th1 responses to anti-inflammatory Th2 activity, rebalancing Th1/Th2 immunity in pregnancy [259]. Calcineurin inhibitors selectively suppress T cell activation by inhibiting IL-2 synthesis, modulating T cell-mediated inflammation and offering disease control in conditions like lupus nephritis [260], [261], [262]. Sulfasalazine reduces reactive oxygen species and inflammatory mediators released by phagocytes, while TNF inhibitors directly block TNF-α signaling, thereby reducing inflammation in both maternal-fetal interface and maternal immune system [186], [263]. In contrast, intravenous immunoglobulin (IVIG) exerts broad immunomodulatory effects, including suppression of B cell activity and enhancement of regulatory pathways [264], [265], [266]. Collectively, these medications help restore immune balance within the maternal-fetal interface, mitigating inflammation-associated pregnancy complications while reducing maternal disease flares.

From a clinical standpoint, the selection of these medications during pregnancy requires careful consideration of pharmacokinetics, placental transfer, and long-term maternal and fetal safety. Physiological changes, such as increased plasma volume, altered hepatic enzyme activity, and enhanced renal clearance, may significantly affect drug distribution, metabolism, and clearance. For instance, corticosteroids and HCQ exhibit dose-dependent pharmacokinetics and increased clearance, necessitating dose adjustments to maintain therapeutic levels while minimizing adverse effects [267]. Placental transfer varies across these drugs. Certolizumab pegol shows minimal transplacental passage, making it safer throughout gestation [268]. In contrast, tacrolimus and azathioprine do cross the placenta, though fetal exposure is significantly lower [269]. Their use must be balanced with risks, including fetal growth restriction (long-term corticosteroids), ductus arteriosus closure [non-steroidal anti-inflammatory drug (NSAID) use in late pregnancy], or hematologic toxicity (high-dose sulfasalazine) [270]. Long-term studies generally support the biosafety of HCQ, certolizumab, and low-dose azathioprine, with no significant increase in congenital anomalies, though careful fetal monitoring and dose optimization are critical [269]. Overall, individualized approach is essential to ensure both maternal disease control and fetal safety.

## 5 Conclusions and outlook

The immune microenvironment in AIDs undergoes dynamic and disease-specific changes during pregnancy, significantly influencing maternal and fetal outcomes. Compared to normal pregnancy, women with AIDs are at greater risk of APOs, largely due to the failure of the typical shift from a proinflammatory to an anti-inflammatory immune phenotype at the maternal-fetal interface. This is especially evident in SLE, APS, SS, and AITD, where reduced Treg levels and antibody-mediated interference with embryo implantation and placental development are common. Conversely, certain AIDs, including RA, MS, and CD, often show clinical improvement, potentially driven by a Th2-skewed immune response, hormonal modulation, and changes in antibody glycosylation. This heterogeneity in immune responses underscores the complexity of pregnancy-related immune adaptations across different AIDs. Moreover, the disease-specific effects of immunomodulatory therapies highlight the limitations of uniform treatment strategies, emphasizing the need for individualized management.

Despite progress, critical gaps remain, the mechanisms underlying pregnancy-induced remission in RA are still unclear, and localized immune alterations in diseases like SLE remain insufficiently characterized. These challenges hinder the development of optimized therapeutic approaches. Advances in single-cell sequencing, epigenetics, and immunomonitoring technologies now offer opportunities to dissect the immunological heterogeneity of AIDs during pregnancy. Integrating these insights into clinical care, through early biomarker identification, precise disease classification, and individualized treatment planning, will advance the management of pregnancy in AIDs. Ultimately, a personalized and dynamically adaptive approach, supported by close collaboration between rheumatologists and obstetricians, is essential for effective management of AIDs.

## Abbreviations

β2GPI: β2-glycoprotein I

ACPAs: Anti-citrullinated protein antibodies

ADCC: Antibody-dependent cellular cytotoxicity

AID: Autoimmune disease

AITD: Autoimmune thyroid disease

aPL: Antiphospholipid

APO: Adverse pregnancy outcome

APS: Antiphospholipid syndrome

AZA: Azathioprine

CCL: Chemokine C-C motif ligand

CD: Crohn’s disease

CHB: Congenital heart block

dNK: Decidua natural killer

dsDNA: Double-stranded DNA

CXCL: C-X-C motif chemokine ligand

ER: Estrogen receptor

EVT: Extravillous trophoblast

FOXP3: Forkhead box P3

HCQ: Hydroxychloroquine

HLA: Human leukocyte antigen

HT: Hashimoto’s thyroiditis

IBD: Inflammatory bowel disease

IFN: Interferon

IL: Interleukin

LIF-R: Leukemia inhibitory factor receptor

LMWH: Low-molecular-weight heparin

IUGR: Intrauterine growth restriction

MMP: Matrix metalloproteinase

MS: Multiple sclerosis

MTX: Methotrexate

NETs: Neutrophil extracellular traps

NK: Natural killer

NLE: Neonatal lupus erythematosus

NSAID: Non-steroidal anti-inflammatory drug

PAPS: Primary antiphospholipid syndrome

PD-L1: Programmed death ligand 1

PE: Preeclampsia

PSG: Pregnancy-specific glycoprotein

pSS: primary Sjögren’s syndrome

RA: Rheumatoid arthritis

SAPS: Secondary antiphospholipid syndrome

SLE: Systemic lupus erythematosus

SS: Sjögren’s syndrome

SSA/Ro: Sjögren’s syndrome-related antigen A

SSB/La: Sjögren’s syndrome-related antigen B

TGF: Transforming growth factor

Th: T helper

TNF: Tumor necrosis factor

Treg: Regulatory T cell

UC: Ulcerative colitis

## Ethics approval and consent to participate

Not applicable.

## Funding

This work was supported by the National Natural Science Foundation of China (82572075, 82270903, 82370465, and 82401588), the Hubei Key Research and Development Project (2023BCB013), the China Postdoctoral Science Foundation (2024M751019), and the Hubei Provincial Hypertension Clinical Medicine Research Center Open Project (HBCH2024002).

## CRediT authorship contribution statement

ZJM and JC wrote the original draft of the manuscript. CXY, SPL, and QG contributed to visualization and investigation. ZB and YYW supervised the study. XQR and JXZ reviewed and edited the manuscript. All authors read and approved the final manuscript.

## Data Availability

Not applicable.
